# Supplementary material for: Sex Differences in Lipid Profile across the Life Span in Patients with Type 2 Diabetes: A Primary Care-Based Study
Source: J Clin Med. 2021 Apr 19;10(8):1775. doi: 10.3390/jcm10081775 (PMC8072568; doi:10.3390/jcm10081775)
Supplement: Supplementary file 1 [file jcm-10-01775-s001.zip › jcm-1167192-conversion SI new.pdf]

Supplementary material

Table S1: Extraction time periods and definitions of variables used in the analyses.

Variable	Time period	Comment
Weight	Last value in five years before or first value in one year after index date	Height and weight were used to calculate BMI
Height	Last value in 10,000 days before or after index date	
BMI[1]	Last value in five years before or first value in one year after index date	BMI was extracted if height and/or weight were missing
HbA1c[1], smoking status, SBP	Last value in 6 months before index date or first value in 30 days after index date	Used as entered in the database
Serum creatinine* and eGFR	Last value in 6 months before index date or first value in 30 days after index date	eGFR was calculated using Modification of Diet in Renal Disease equation or extracted from the database[2] when serum creatinine was missing
Albumin creatinine ratio and albuminuria concentration	Last value in 6 months before index date or first value in 30 days after index date	Albuminuria was defined as albumin creatinine ratio ≥30 mg/g or albuminuria concentration ≥300 mg
Diabetes duration	On index date	
Polypharmacy and glucose lowering treatment	Based on prescriptions in the 3 months before index date	Polypharmacy was defined as a prescription for five or more drugs
History of CVD	Any recording of CVD before index date.	History of CVD included the presence of angina pectoris, acute myocardial infarction, transient ischemic attack, stroke, atherosclerosis, other ischemic heart diseases and peripheral arterial diseases, abdominal aortic aneurysm, percutaneous transluminal (coronary) angioplasty, and peripheral or coronary bypass.

*The standard way of measuring serum creatinine was directly with enzymatic colorimetric methods (Roche elecsys C Module; Roche diagnostics, Switzerland).

[1] Multiple imputation by chained equation was used to impute BMI and HbA1c missing values. The model included LDL-c, HDL-c, TC, TG, and sex. No auxiliary variables were found. We conducted 20 imputations and the results of different datasets were combined by Stata using Rubin's combination rules.

[2] eGFR in the database was calculated using the Modification of Diet in Renal Disease equation.

Table S2: Patient demographics in those not treated and treated with a statin per sex and age group.

			Number of patients	Diabetes duration; median	HbA1c; median	BMI in kg/m²; mean	SBP in mmHg; mean	eGFR ≤60 mL/min/1.73m²; N (%)	Albuminuria; N (%)	Polypharmacy; N (%)	Smoking; N (%)	History of CVD; N(%)
Not treated with a statin	Men	<40	144	1.0	7.1	32.1	133	0 (0)	2 (3)	7 (5)	29 (50)	2 (1)
		40-44	214	1.0	6.9	31.7	137	2 (1)	2 (2)	24 (11)	33 (42)	10 (5)
		45-49	369	0.9	6.8	31.1	138	1 (0)	3 (2)	47 (13)	40 (34)	21 (6)
		50-54	534	1.4	6.8	30.8	138	5 (1)	4 (2)	95 (18)	82 (38)	47 (9)
		55-59	684	2.0	6.7	29.7	140	12 (2)	8 (3)	121 (18)	67 (27)	77 (11)
		60-64	913	2.6	6.7	29.5	143	28 (3)	8 (2)	203 (22)	83 (25)	144 (16)
		65-69	826	2.9	6.7	29.6	145	50 (7)	10 (3)	187 (23)	59 (20)	155 (19)
		70-74	684	3.0	6.7	28.3	146	81 (13)	12 (4)	182 (27)	52 (21)	181 (26)
		75-79	577	3.9	6.7	28.4	147	109 (21)	14 (5)	226 (39)	31 (16)	159 (28)
		80-84	361	4.0	6.7	27.5	144	115 (35)	6 (4)	143 (40)	19 (14)	124 (34)
		≥85	243	5.6	6.8	26.3	145	84 (36)	13 (11)	126 (52)	10 (11)	94 (39)
	Women	<40	150	1.3	7.0	34.4	130	0 (0)	1 (1)	24 (16)	13 (25)	4 (3)
		40-44	214	1.2	6.6	33.3	132	4 (2)	1 (1)	35 (16)	15 (18)	10 (5)
		45-49	350	1.0	6.7	33.6	136	5 (2)	0 (0)	75 (21)	35 (26)	41 (12)
		50-54	494	1.4	6.8	32.7	139	17 (4)	2 (1)	129 (26)	75 (39)	46 (9)
		55-59	603	1.5	6.7	31.8	138	26 (5)	3 (2)	148 (25)	69 (28)	53 (9)
		60-64	806	2.2	6.7	31.0	142	51 (7)	3(1)	229 (28)	66 (20)	99 (12)
		65-69	781	2.9	6.7	30.8	146	82 (12)	6 (2)	240 (31)	54 (19)	128 (16)
		70-74	808	3.1	6.7	30.2	146	121 (17)	5 (2)	335 (41)	54 (19)	167 (21)
		75-79	788	4.0	6.7	29.8	146	176 (24)	14 (5)	351 (45)	41 (14)	192 (24)
		80-84	705	4.8	6.7	29.0	146	250 (38)	10 (4)	363 (51)	18 (8)	203 (29)
		≥85	707	7.4	6.8	28.1	145	317 (47)	19 (7)	406 (67)	21 (9)	200 (28)

			Number of patients	Treated with a statin out of all in age group (%)	Diabetes duration; median	HbA1c; median	BMI in kg/m^2; mean	SBP in mmHg; mean	eGFR ≤60 mL/min/1.73m^2; N (%)	Albuminuria; N (%)	Polypharmacy; N (%)	Smoking; N (%)	History of CVD; N(%)
Treated with a statin	Men	<40	70	33	3.2	6.7	33.2	132	0 (0)	2 (5)	16 (23)	10 (29)	4 (6)
		40-44	157	42	2.9	6.9	31.8	134	2 (1)	0 (0)	59 (38)	32 (43)	17 (11)
		45-49	308	45	2.9	6.8	31.7	136	1 (0)	3 (2)	117 (38)	54 (47)	44 (14)
		50-54	628	54	3.7	6.8	30.8	138	13 (2)	6 (2)	295 (47)	99 (38)	122 (19)
		55-59	934	58	4.3	6.9	30.6	138	27 (3)	11 (2)	476 (51)	113 (33)	251 (27)
		60-64	1,402	61	4.5	6.8	30.1	140	56 (4)	27 (4)	770 (55)	183 (30)	425 (30)
		65-69	1,359	62	4.7	6.8	29.8	144	109 (9)	24 (3)	797 (59)	137 (25)	490 (36)
		70-74	1,139	62	4.8	6.8	28.9	142	156 (15)	24 (4)	733 (64)	106 (22)	503 (44)
		75-79	878	60	5.5	6.8	28.7	143	199 (24)	24 (5)	628 (72)	65 (19)	438 (50)
		80-84	479	57	5.9	6.8	28.1	143	159 (35)	15 (6)	360 (75)	29 (15)	261 (54)
		≥85	213	47	6.9	6.9	27.1	142	80 (40)	10 (10)	169 (79)	9 (10)	127 (60)
	Women	<40	53	26	2.7	6.7	35.9	129	0 (0)	0 (0)	21 (40)	9 (43)	5 (9)
		40-44	113	35	2.7	6.8	34.3	131	0 (0)	1 (2)	54 (48)	13 (25)	19 (17)
		45-49	266	43	3.5	6.8	34.5	134	6 (3)	1 (1)	133 (50)	35 (30)	40 (15)
		50-54	445	47	3.8	6.8	32.6	135	13 (3)	8 (4)	241 (54)	73 (39)	68 (15)
		55-59	701	54	4.2	6.8	31.7	138	40 (6)	9 (3)	389 (55)	78 (27)	134 (19)
		60-64	1,068	57	4.6	6.7	31.3	140	85 (9)	13 (3)	627 (59)	122 (27)	214 (20)
		65-69	1,237	61	5.1	6.8	30.9	143	143 (13)	11 (2)	782 (63)	107 (21)	321 (26)
		70-74	1,146	59	5.5	6.8	30.6	144	201 (19)	20 (4)	794 (69)	86 (19)	353 (31)
		75-79	1,116	59	6.0	6.8	29.8	145	311 (30)	23 (5)	800 (72)	63 (14)	363 (33)
		80-84	770	52	7.3	6.8	29.2	147	273 (38)	21 (6)	599 (78)	33 (12)	309 (40)
		≥85	412	37	9.2	6.9	28.1	147	161 (41)	11 (6)	340 (83)	11 (7)	183 (44)

Table S3: Sex differences in lipid levels across age groups adjusted for BMI in those (A) not treated and (B) treated with a statin. Cholesterol measurements are in mmol/L.

**A – Not treated with a statin**		<40	40-44	45-49	50-54	55-59	60-64	65-69	70-74	75-79	80-84	≥85
LDL-c; mean±SE	Men	3.3±.08	3.4±.06	3.3±.05	3.4±.04	3.3±.04	3.2±.03	3.3±.03	3.2±.04	3.1±.04	3.1±.05	2.9±.06
	Women	3.1±.08	3.1±.06	3.3±.05	3.5±.04	3.5±.04	3.6±.03	3.5±.03	3.4±.03	3.3±.03	3.3±.04	3.2±.04
	p	**0.039**	**0.004**	0.701	**0.012**	**<0.001**	**<0.001**	**<0.001**	**<0.001**	**<0.001**	**0.002**	**<0.001**
HDL-c; mean±SE	Men	1.0±.03	1.1±.02	1.1±.02	1.1±.01	1.2±.01	1.2±.01	1.2±.01	1.2±.01	1.2±.01	1.2±.02	1.2±.02
	Women	1.2±.03	1.3±.02	1.3±.02	1.3±.02	1.4±.01	1.4±.01	1.4±.01	1.4±.01	1.4±.01	1.4±.01	1.3±.01
	p	**<0.001**	**<0.001**	**<0.001**	**<0.001**	**<0.001**	**<0.001**	**<0.001**	**<0.001**	**<0.001**	**<0.001**	**<0.001**
TG; mean±SE	Men	2.4±.09	2.5±.08	2.5±.06	2.3±.05	2.0±.04	1.9±.04	1.8±.04	1.7±.04	1.8±.05	1.6±.06	1.6±.07
	Women	1.7±.09	1.7±.08	1.8±.06	1.9±.05	1.8±.05	1.8±.04	1.8±.04	1.8±.04	1.8±.04	1.8±.04	1.7±.04
	p	**<0.001**	**<0.001**	**<0.001**	**<0.001**	0.068	0.564	0.645	0.056	0.687	0.073	0.475
TC; mean±SE	Men	5.3±.09	5.4±.07	5.3±.06	5.4±.05	5.2±.04	5.1±.04	5.1±.04	5.0±.04	5.0±.05	4.9±.06	4.7±.07
	Women	5.0±.09	5.0±.07	5.2±.06	5.5±.05	5.6±.04	5.6±.04	5.6±.04	5.5±.04	5.4±.04	5.3±.04	5.1±.04
	p	**0.030**	**0.001**	0.259	**0.008**	**<0.001**	**<0.001**	**<0.001**	**<0.001**	**<0.001**	**<0.001**	**<0.001**
Non-HDL-c; mean±SE	Men	4.2±.09	4.3±.07	4.2±.06	4.2±.05	4.0±.04	3.9±.04	4.0±.04	3.8±.04	3.8±.05	3.7±.06	3.5±.07
	Women	3.8±.09	3.7±.07	3.9±.06	4.2±.05	4.2±.04	4.2±.04	4.1±.04	4.1±.04	4.0±.04	3.9±.04	3.8±.04
	p	**<0.001**	**<0.001**	**<0.001**	0.591	**<0.001**	**<0.001**	**0.001**	**<0.001**	**0.001**	**0.002**	**<0.001**
**B – Treated with a statin**												
LDL-c; mean±SE	Men	2.3±.09	2.4±.06	2.4±.04	2.4±.03	2.3±.02	2.3±.02	2.2±.02	2.2±.02	2.1±.02	2.0±.03	2.0±.05
	Women	2.4±.10	2.4±.07	2.4±.05	2.4±.03	2.4±.03	2.4±.02	2.4±.02	2.4±.02	2.3±.02	2.2±.03	2.1±.04
	p	0.534	0.820	0.767	0.747	**0.004**	**<0.001**	**<0.001**	**<0.001**	**<0.001**	**<0.001**	0.116
HDL-c; mean±SE	Men	1.1±.04	1.1±.03	1.1±.02	1.1±.01	1.1±.01	1.2±.01	1.2±.01	1.2±.01	1.2±.01	1.2±.01	1.2±.02
	Women	1.3±.04	1.2±.03	1.3±.02	1.3±.02	1.3±.01	1.4±.01	1.4±.01	1.4±.01	1.4±.01	1.4±.01	1.4±.02
	p	**0.001**	**<0.001**	**<0.001**	**<0.001**	**<0.001**	**<0.001**	**<0.001**	**<0.001**	**<0.001**	**<0.001**	**<0.001**
TG; mean±SE	Men	2.2±.12	2.4±.08	2.2±.06	2.1±.04	1.9±.03	1.8±.03	1.7±.03	1.6±.03	1.6±.03	1.6±.05	1.5±.07
	Women	1.8±.14	1.7±.10	1.8±.06	1.8±.05	1.8±.04	1.8±.03	1.7±.03	1.7±.03	1.6±.03	1.6±.04	1.6±.05
	p	**0.028**	**<0.001**	**<0.001**	**<0.001**	**0.006**	0.867	0.158	0.089	0.143	0.491	0.254
TC; mean±SE	Men	4.1±.11	4.3±.07	4.3±.05	4.2±.04	4.2±.03	4.1±.02	4.0±.02	4.0±.03	3.9±.03	3.8±.04	3.7±.06
	Women	4.3±.12	4.2±.08	4.3±.05	4.4±.04	4.4±.03	4.5±.03	4.4±.03	4.4±.03	4.3±.03	4.2±.03	4.1±.04
	p	0.385	0.811	0.567	**0.024**	**<0.001**	**<0.001**	**<0.001**	**<0.001**	**<0.001**	**<0.001**	**<0.001**
Non-HDL-c; mean±SE	Men	3.1±.10	3.2±.07	3.2±.05	3.1±.03	3.0±.03	2.9±.02	2.9±.02	2.8±.03	2.7±.03	2.6±.04	2.5±.06
	Women	3.0±.12	3.0±.08	3.0±.05	3.0±.04	3.1±.03	3.1±.03	3.0±.02	3.0±.03	2.9±.03	2.8±.03	2.7±.04
	p	0.707	0.063	**0.006**	0.059	0.141	**<0.001**	**<0.001**	**<0.001**	**<0.001**	**<0.001**	**0.044**

p<0.05 was considered statistically significant (marked bold); SE = standard error; LDL-c = low-density lipoprotein cholesterol; HDL-c = high-density lipoprotein cholesterol; TG = triglycerides; TC = total cholesterol

(A)

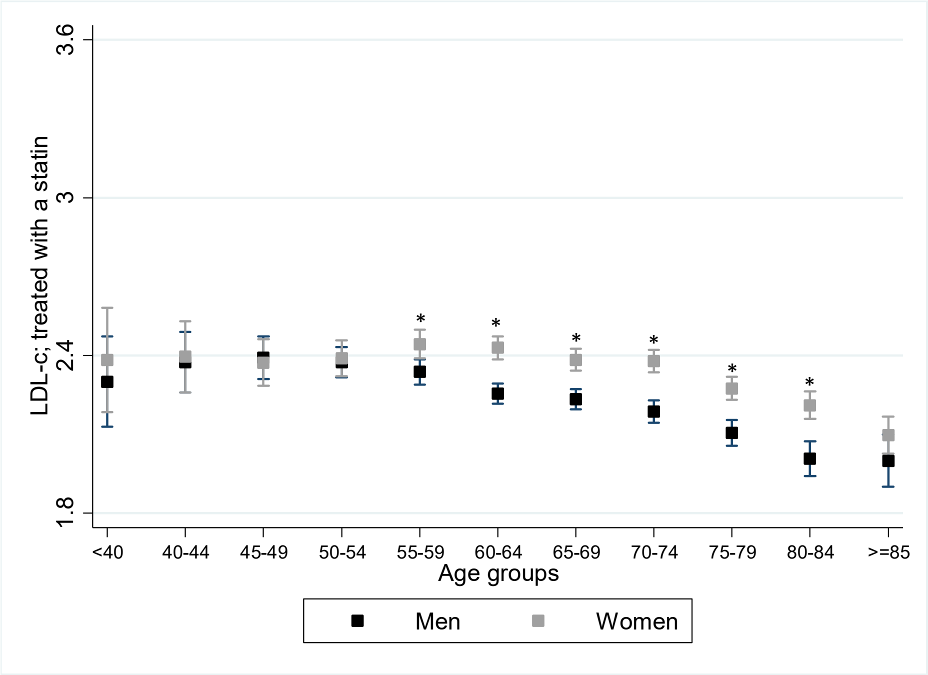
LDL-c; treated with a statin
1.8
2.4
3
3.6
*
<40
40-44
45-49
50-54
55-59
60-64
65-69
70-74
75-79
80-84
>=85
Age groups
Men
Women

(B)

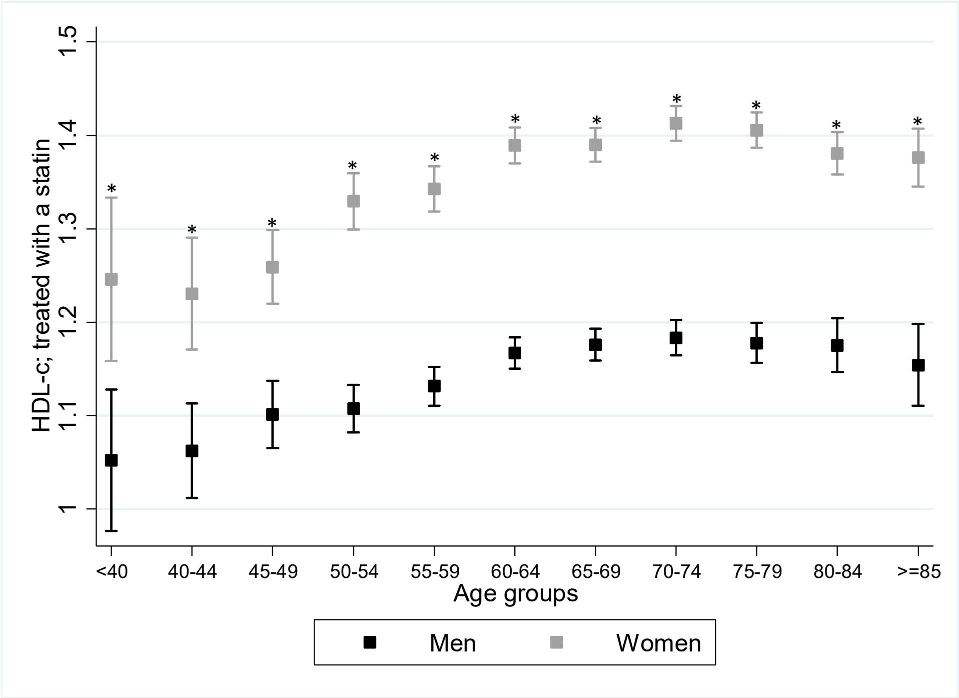
HDL-c; treated with a statin
1
1.1
1.2
1.3
1.4
1.5
*
<40
40-44
45-49
50-54
55-59
60-64
65-69
70-74
75-79
80-84
>=85
Age groups
Men
Women

(C)

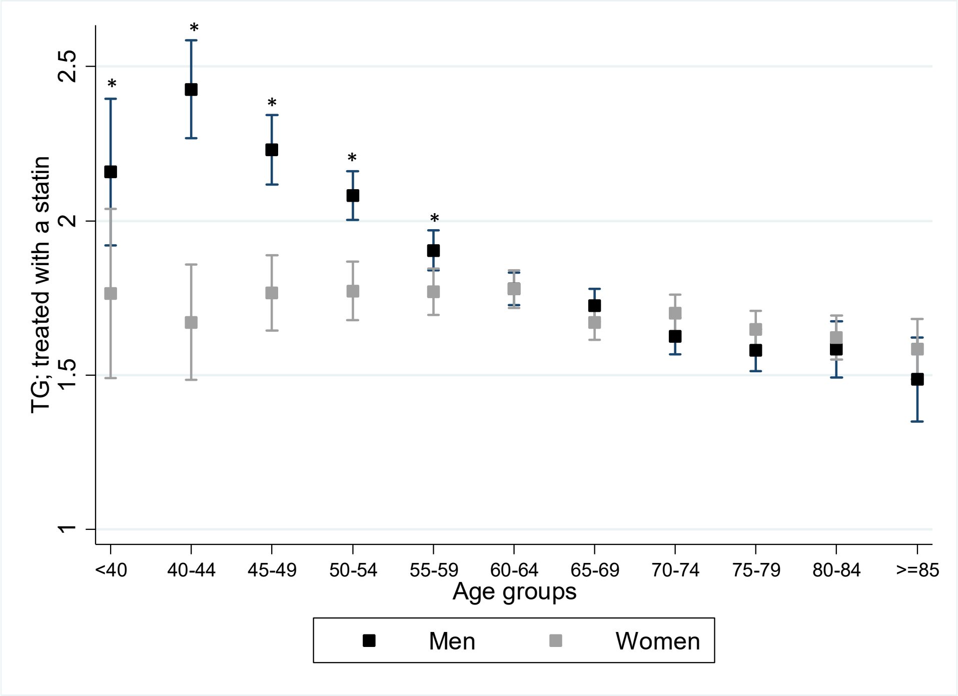

Figure S1: Mean lipid levels with 95% CIs for men and women per age group treated by a statin of (A) low-density lipoprotein cholesterol (LDL-c), (B) high-density lipoprotein cholesterol (HDL-c) and (C) triglycerides (TG). Cholesterol measurements are in mmol/L. Values are adjusted for body mass index and statin dose (moderate versus high; dose was not available for 8 patients); TG values are additionally adjusted for glycated hemoglobin A1c. *p<0.05 between men and women.

(A)

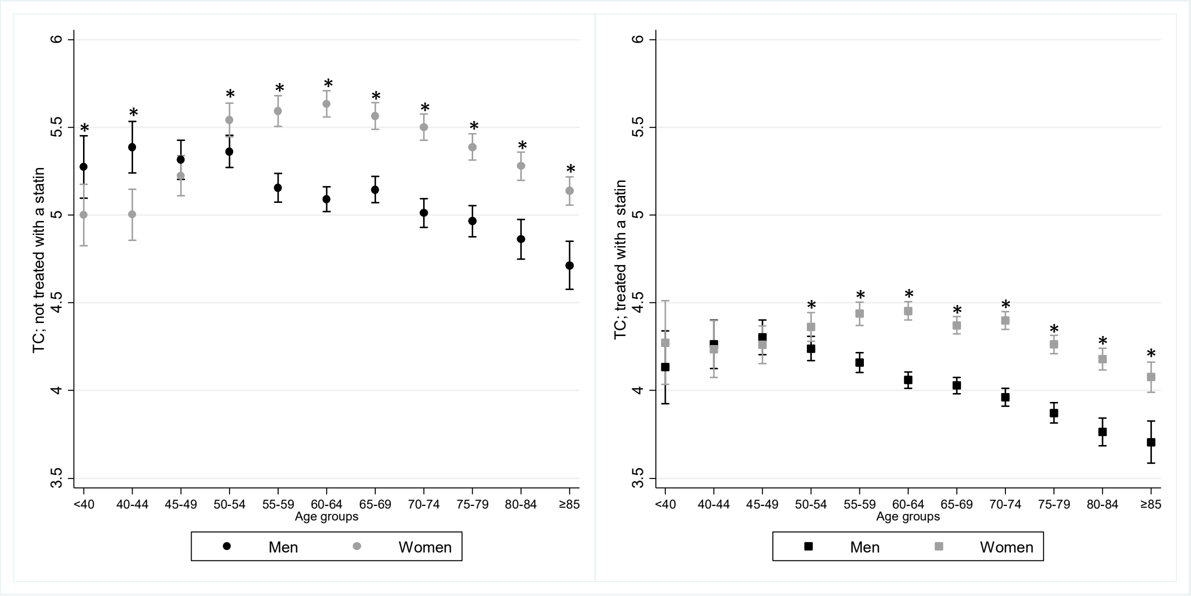

(B)

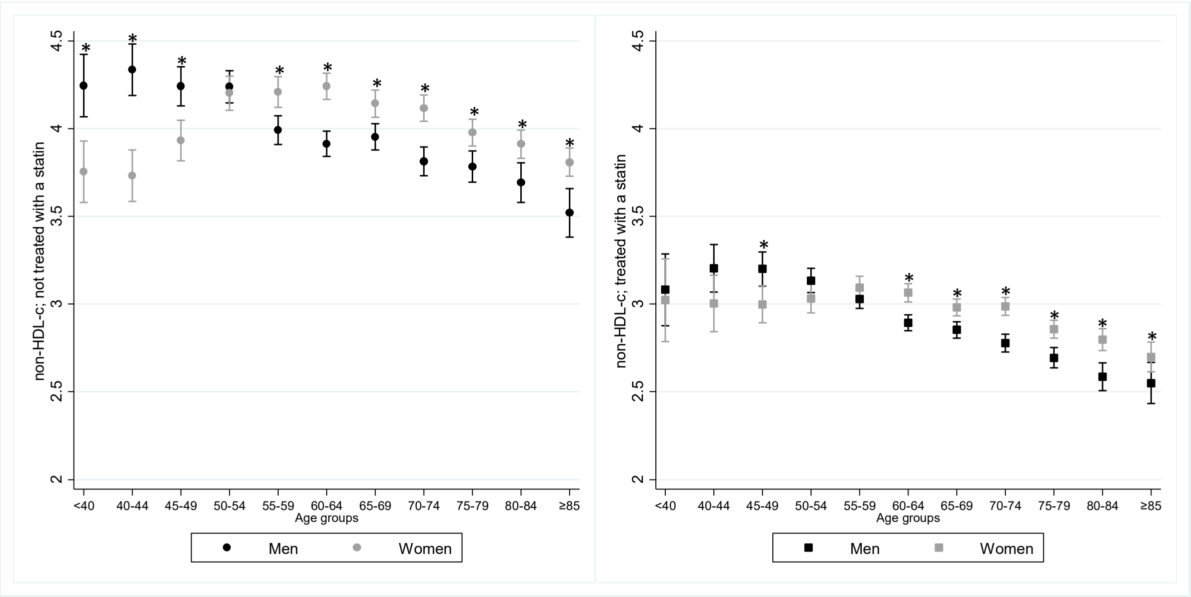

Figure S2: Mean lipid levels with 95% CIs for men and women per age group in those not treated (left) and treated with a statin (right) of (A) total cholesterol (TC) and (B) non-HDL cholesterol. Cholesterol measurements are in mmol/L. All values are adjusted for body mass index (BMI); TG values are additionally adjusted for glycated hemoglobin A1c. *$p<0.05$ between men and women.
